# Microelements and macroelements in the body of the invasive Harris mud crab (*Rhithropanopeus harrisii*, Maitland, 1874) from the central coast of the South Baltic Sea

**DOI:** 10.1007/s10661-019-7564-3

**Published:** 2019-07-18

**Authors:** Arkadiusz Nędzarek, Przemysław Czerniejewski, Agnieszka Tórz

**Affiliations:** 1 Department of Aquatic Sozology, Faculty of Food Sciences and Fisheries, West Pomeranian University of Technology in Szczecin, Kazimierza Królewicza Street 4, 71-550 Szczecin, Poland; 2 Department of Fisheries Management, Faculty of Food Sciences and Fisheries, West Pomeranian University of Technology in Szczecin, Kazimierza Królewicza Street 4, 71-550 Szczecin, Poland

**Keywords:** Crustaceans, Exoskeleton, Feed fish, Essential metals, Heavy metals, Arsenic

## Abstract

In this study, we determined the levels of essential and non-essential elements in the Harris mud crab (*Rhithropanopeus harrisii*, Maitland, 1874) from the southern Baltic Sea. Results revealed high levels of Ca (246,000 ppm), Mg (11,000 ppm), Na (8160 ppm), K (3,780 ppm), and Fe (1830 ppm). The concentrations of essential metals such as Zn (62.5 ppm) and Cu (25 ppm) were similar to those recorded in other crab species. The concentrations of non-essential metals such as Pb (0.140 ppm), Cd (0.0017 ppm), and As (0.288 ppm) were well below the International Standards for Maximum Levels for Food. In view of the above, the Harris mud crab from the southern Baltic is safe to be used as a component of well-balanced feeds for terrestrial and aquatic animals.

## Introduction

The natural habitat of the Harris mud crab (*Rhithropanopeus harrisii*) is the brackish estuarine waters of the Atlantic coast, stretching from the northern regions of the Gulf of Mexico to Nova Scotia in Canada (Petersen 2006). In Europe, the first specimens of this species were observed in 1874 in the Zuiderzee River (Netherlands), and in 1936 their presence was confirmed in the Baltic Sea (Buitendijk and Holthuis 1949). Currently, the Harris mud crab has been reported as a non-indigenous species in 21 different countries (Roche and Torchin 2007). In Poland, the Harris mud crab was first recorded in the 1950s in the Vistula Lagoon (Demel 1953), the estuary of the Vistula, and the Gulf of Gdańsk (Kujawa 1957; Turoboyski 1973; Normant et al. 2004).

Research on crab biology in the Gulf of Gdańsk area began with Michalski (1957) and Kujawa (1957), and in the western part of the Baltic Sea by Czerniejewski and Rybczyk (2008) and Czerniejewski (2009). Analyses by Turoboyski (1973), Normant et al. (2004), and Czerniejewski (2009) showed that these small crustaceans, with a carapace width not exceeding 27 mm, are euryhaline animals that mainly feed on detritus and fine zoobenthos. They are preyed upon by fish which are able to regulate their populations in many waters (Turoboyski 1973; Fowler et al. 2013).

The Harris mud crab may be one of the vectors of heavy metal transfer in the trophic pyramid in the coastal zone of the southern Baltic Sea, with significant amounts of these elements being supplied by river systems (Szefer 2002). Metals present in the environment are ingested by crabs together with food (from food itself, sediment particles, and water), directly from the water through the respiratory system, and also as a result of adsorption through the carapace (e.g., Nędzarek et al. 2017, 2019). Bottom sediments play a particularly important role here. The bottom sediments in which the crabs live accumulate pollutants and therefore constitute a secondary source in the ecosystem (Fang et al. 2016; Ke et al. 2017). These sediments provide food and a habitat to benthic organisms, which are then eaten by fish (Demirak et al. 2006; Vicente-Martorell et al. 2009). As a consequence, the quality and safety of seafood may be at risk (Turkmen et al. 2005, Peng et al. 2011).

Crustaceans are a valuable source of high-quality protein, fatty acids, and macroelements and microelements such as K, P, Na, Ca, Mg, Cu, Fe, and Zn (Moronkola et al. 2011; Erdem et al. 2015; Islam et al. 2016). However, the Harris mud crab is a relatively small crustacean, and too small for human consumption, but may be used as an additive to fish products used in animal feed production (Jimmy and Arazu 2012). The aforementioned elements are those that are essential for the proper development of organisms. However, organisms may also accumulate non-essential toxic elements (e.g., Hg, Cd, Pb, As). These elements can permanently enter the food chain and are particularly dangerous for human health. Therefore, they are used as environmental quality indicators and their concentration in food is controlled by various international regulations (e.g., Standard of Maximum Levels of Contaminants in Foods, FAO 2003, Commission Regulation (EC) No. 1881/2006; Council Directive 98/83/EC 1998). Cd, Pb, and As concentrations are also regulated in raw material for animal feed (Directive 2002/32/EC). Therefore, it is important to know and evaluate the levels of elements in the raw material, including heavy metals or other potentially toxic elements such as arsenic.

To date, the population of Harris mud crabs inhabiting the southern Baltic has not been examined in the context of levels of macroelements and microelements, such as Ca, Mg, Na, K, Fe, Zn, Cu, Al, Ni, As, Pb, and Cd. Therefore, the aim of this research was to assess the content of essential and non-essential elements in the Harris mud crab from the central part of the South Baltic coast against the background of environmental conditions (water and bottom sediments).

## Material and methods

### Study area

**Fig. 1:**
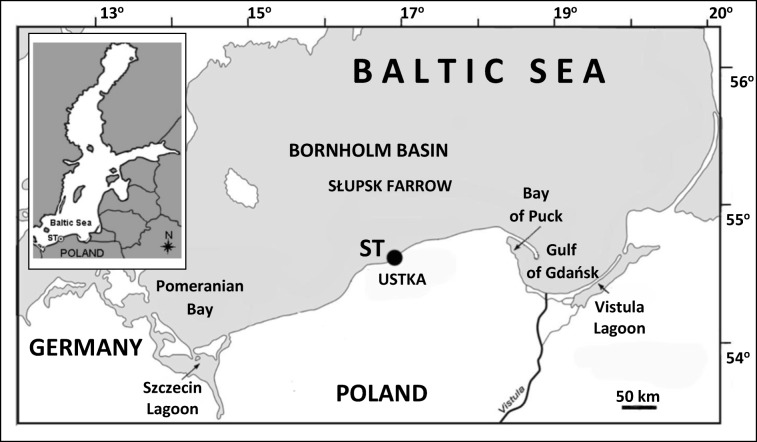
The area of sampling water, bottom sediments, and Harris mud crabs from the southern coast of the Baltic Sea, (ST-station)

The research area covered the southern coast of the Baltic Sea to a depth of 5 m near the seaport in Ustka (E 16° 50′ 59.7″, N 54° 35′ 30″) (Fig. 1). According to HELCOM CORESET BD 2/2011 (Helcom 2011), this is part of sub-basin no. 38, also known as the Polish coastal waters of the Bornholm Basin. These waters are characterized by moderate annual temperature (13.1 °C), low salinity (7.1 PSU), sandy bottom, and intense mixing of the waters (Krzymiński 2014). This last factor, together with sea currents, causes the dispersion and transfer of small material fractions to deeper areas (so-called accumulation areas). According to Szefer (2002), heavy metals exhibiting affinity for fine particles and organic matter do not accumulate in these shallow sediments, which results in lower concentrations of heavy metals found in bottom sediments in this area compared with deeper Baltic sub-basins (Uścinowicz et al. 2011).

The main source of pollution in the research area is the Ustka seaport located in the estuary of the Słupia River. Antonowicz et al. (2017) showed extremely high heavy metal concentrations in the central area of the port, with lower concentrations in the outport and in the upstream Słupia River. Likewise, the concentration of heavy metals in sediments at nearby beaches decreased with the distance from the port.

### Sampling procedure

Twenty-four (24) Harris mud crabs were caught between September 22 and November 6, 2016, using a drag net, off the coast of the central Baltic Sea (near the town of Ustka) (Fig. 1). Each crab was properly cleaned by rinsing with water to remove debris, plankton, and other external adherents. Immediately after sampling, the crabs were stored in a container placed in crushed ice for transport to the laboratory, where they were frozen at − 20 °C until analysis.

Water samples were taken at 3 sites (4 samples from each site) near the places where the crabs were caught. Pre-cleaned (rinsed at least three times) 1,5-l polyethylene sampling bottles were immersed about 0.5 m below the water surface, then stored in a cooler box for transport to the laboratory. Seabed sediment samples (0–10 cm) were also taken using a Peterson grab at the same 3 sites (4 samples from each site), kept in ice for transport to the laboratory, then packed in polyethylene bags and frozen at − 20 °C until analysis. All the samples were kept away from metallic materials to avoid contamination.

After thawing and drying to a constant weight at 90 °C, the whole body (including meat and exoskeleton) of crabs were crushed in an agate mortar. 1 g ± 0.01 samples were then digested in 10 mL of concentrated ultrapure HNO_3_ (Merck, Germany) in a high-pressure microwave digestor, Speedwave Xpert (Bergoff, Eningen Germany), then diluted with Milli-Q water (18.2 MΩ) to 25 mL.

Samples of the habitat sea water were digested with concentrated HNO_3_ at a ratio of 10:1 sample to acid by volume. Seabed sediments, after thawing and drying to a constant weight at 90 °C, were filtered through a 2-mm sieve, then wetted with deionized water. The sediments were then dried to a constant weight at 90 °C and digested with concentrated HNO_3_ at a ratio of 2 g of sediment to 5 ml of HNO_3_ (digestion time, 30 min; temperature, 200 °C). The resulting solution was diluted with deionized water to 25 mL.

Elements were determined using a Hitachi Polarized Zeeman atomic absorption spectrometer ZA3000 series (Hitachi High-Technologies Corporation, Tokyo, Japan). Ca, K, Na, and Mg were determined by flame method (FAAS) in an air-acetylene flame. The concentrations of Al, As, Cd, Cu, Fe, Ni, Pb, and Zn were measured using a flameless graphite furnace (GFAAS). According to the analytical methodology presented by the APHA (2005), dedicated matrix modifiers were used for selected elements, such as palladium in 5% HNO_3_ (Sigmatik, Poland) and cesium chloride-lanthanum chloride buffer solution acc. to Schinkel (Merck, Germany).

Calibration curves were made using certified standard solutions (1000 mg/L) from Scharlau (Spain) for Mg, Ca, K, Na, and Fe, and from Merck (Germany) for Al, As, Cd, Cu, Ni, Pb, and Zn. The detection limits were 0.3 ppb (for Al), 0.2 ppb (for Cd and Pb), 0.5 ppb (for Fe and Ni), 0.1 ppb (for Zn), 2.0 ppb (for As), and 1.0 ppb (for Cu).

The analytical method was tested using reference fish muscle ERM-BB422 (European Reference Materials, European Commission–Joint Research Center, Institute for Reference Materials and Measurements, Geel, Belgium). The recovery of elements ranged from 95 to 105%, and the precision for the reference materials ranged from 1.2 to 10.1% (Table 1).

**Table 1:** Recovery of elements from the reference material (fish muscle ERM-BB422)

Element	Certified value	Determined value (*N* = 3)		Recovery	SD
	ppm		SD	%	
Ca	3420	3487	61	102	1.8
K	21,400	21,643	895	101	4.2
Mg	1370	1304	17	95	1.2
Na	2800	2937	91	105	3.2
Fe	9.4	9.0	0.3	96	3.4
Cu	1.67	1.69	0.09	101	5.4
Zn	16	16.8	0.8	105	1.6
Cd	0.0075	0.0072	0.0008	96	10.1

### Statistical analysis

Statistical analysis was performed using Statistica v12.0 (StatSoft Inc 2016). Means and standard deviations (SD) were determined. Differences in the accumulation of elements between the Harris mud crab, water, and sediments were calculated using a Kruskal-Wallis test. The level of significance for the statistical tests was *p* < 0.05.

## Results and discussion

The Harris mud crab is the smallest invasive crab species of the genus *Brachyura* in the Baltic Sea (Czerniejewski and Rybczyk 2008; Hegele-Drywa and Normant 2014). In the eastern part of the Polish Baltic coast, the width of its carapace ranges from 1.96 to 21.4 mm (Hegele-Drywa and Normant 2014; Hegele-Drywa et al. 2014), and in the western part from 5.6 to 22.9 mm (Czerniejewski and Rybczyk 2008; Czerniejewski 2009). In the results presented by Normant et al. (2004) and Roche and Torchin (2007), crabs caught on the central coast of the southern Baltic were quite large in size (the width of the carapace was from 13 to 23 mm), which indicates that they were mature and ready for breeding. The lack of smaller individuals in the samples resulted from the parameters of the equipment used for fishing. The average carapace width of the individuals in our study (15 mm, Table 2) matched the populations studied in Czerniejewski and Rybczyk (2008) and Czerniejewski (2009).

**Table 2:** Dry weight, length, and width of the Harris mud crab (*Rhithropanopeus harrisii*) and the levels of elements in the body (*n* = 24)

Indicator		Mean	Minimum	Maximum	SD
Dry weight	g	0.936	0.300	2.254	0.426
Width	mm	18.0	13.0	23.0	2.19
Length		15.0	10.0	20.0	2.05
Ca	ppm d.w.	246,000	229,000	275,000	10,000
Mg		11,000	8680	12,900	935
Na		8610	8080	9490	416
K		3780	2830	4310	284
Fe		1830	1040	2590	366
Al		184	111	330	67
Zn		62.6	51.5	87.6	7.7
Cu		25.0	18.4	37.3	4.8
Ni		0.543	0.378	1.042	0.181
As		0.288	0.210	0.436	0.063
Pb		0.140	0.108	0.238	0.029
Cd		0.0017	0.0003	0.0070	0.0019

*SD*, standard deviation

In our study, the concentrations of the elements determined in the whole body (including meat and exoskeleton) of the Harris mud crab could be arranged in the following descending order: Ca > Mg > Na > K > Fe > Al > Zn > Cu > Ni > As > Pb > Cd (Table 2). That order was different in the surrounding water (Na > Mg > Ca > K > Zn > Cu > Fe > Al > Ni > Pb > As>Cd) and bottom sediments (Ca > Fe > Al > Mg > K > Na > Zn > Cu > Ni > Pb > As>Cd) (Table 3). The concentrations of all elements were significantly higher in the crabs than in the water, with Ca, Mg, Na, K, Fe, Zn, and Cu significantly higher than in the bottom sediments (Table 4). Such a disparity may indirectly indicate the level of contamination of the studied area, especially with non-essential elements. The Baltic Sea is known to be exposed to high levels of anthropogenic stress due to its location and hydrology (Hendożko et al. 2010; Szefer et al. 2009). However, the highest levels of pollution of the southern coast of this sea, including contamination with heavy metals, are found in the area of the Vistula and Odra estuaries. For example, in the sediments of the Bay of Gdańsk, Szefer et al. (2009) recorded As > 4 ppm, Pb > 30 ppm, and Cd > 0.5 ppm. Similarly high concentrations of these elements were also recorded in sediments from the Pomeranian Bay or Słupsk Farrow. The significantly higher concentrations of non-essential elements observed by Szefer et al. (2009) and Hendożko et al. (2010), in comparison with our results, may be due to varying physicochemical properties of sediments across the Baltic Sea coast. The Bay of Gdańsk and Pomeranian Bay are areas which accumulate contaminants and terrigenous material (including organic matter) transported by the Vistula and Odra rivers, respectively. The central-southern Baltic coast is beyond the direct inflow of such high loads of anthropogenic pollution, and the bottom sediments of this area consist mainly of sand, which accumulates less metals than organic material, an important component of the sediments studied by Szefer et al. (2009) and Hendożko et al. (2010).

**Table 3:** Concentration of elements in the water and bottom sediments from the sampling area (*n* = 9)

Element		Water		Sediments	
		Mean	SD	Mean	SD
Ca	ppm	126	5	5122	675
Mg		270	32	402	11
Na		1868	82	52	23
K		77.2	1.8	172	24
Fe		0.502	0.245	1429	180
Al		0.257	0.179	645	124
Zn		1.217	0.281	2.741	0.186
Cu		0.517	0.116	1.672	0.492
Ni		0.0031	0.0016	1.264	0.204
As		0.0009	0.0004	0.292	0.015
Pb		0.0014	0.0002	0.748	0.071
Cd		0.0003	0.0001	0.0068	0.0008

*SD*, standard deviation

**Table 4:** The Kruskal-Wallis test for the significance of elemental differences in the Harris mud crab (HMC), water, and bottom sediments

Element		Water	Bottom sediments	Element		Water	Bottom sediments
Ca	HMC	0.00000*	0.00174*	Zn	HMC	0.00000*	0.00174*
	Water		0.35894		Water		0.35894
Mg	HMC	0.00000*	0.00173*	Cu	HMC	0.00000*	0.00174*
	Water		0.35894		Water		0.35894
Na	HMC	0.00000*	0.00000*	Ni	HMC	0.00174*	0.00174*
	Water		0.35894		Water		0.00000*
K	HMC	0.00000*	0.00173*	As	HMC	0.00006*	1.00000
	Water		0.35894		Water		0.00035*
Fe	HMC	0.00001*	0.02957*	Pb	HMC	0.00174*	0.00174*
	Water		0.11395		Water		0.00000*
Al	HMC	0.00174*	0.00174*	Cd	HMC	0.01571*	0.00174*
	Water		0.00000*		Water		0.00001*

*Statistically significant, *p* < 0.05

As in the conclusions of Hendożko et al. (2010), the lower concentrations of Al, Ni, Cd, and Pb in crabs than in sediments found in our study could be due to the low lability of these metals and consequently their low bioavailability to crabs. Although the influence of sediment geochemistry on the bioavailability of heavy metals is not yet fully understood, it is known that it depends on, among others, the binding of elements with fine sand particles, binding force, redox potential, salinity of water, or type of solid phase (Marmolejo-Rodriquez et al. 2007). Nevertheless, it is known that for benthic organisms, including crabs, metals that are easily extracted from sediments or dissolved in water are particularly bioavailable (Hendożko et al. 2010).

Generally, it can be concluded that the very high concentrations of Ca and Mg observed in the Harris mud crabs in our study (246,000 and 11,000 ppm respectively) were mainly the levels of these elements in their exoskeletons than in the flesh. This is particularly true for Ca, which in the form of calcium carbonate, in addition to chitin and proteins, is the main constituent of the crustacean exoskeleton structure (Pires et al. 2017). For example, in the study by Boßelmann et al. (2007), the exoskeletons of crustaceans *Maja squinado* contained 17.3–27.8% Ca and 1–1.4% Mg, while Pires et al. (2017) found from about 30.8 to about 56.6% Ca in *Cancer pagurus*. Fulton and Fairchild (2013), in their analysis of the whole body of *Carcinus maenas* found much lower levels, 5.7% Ca and 0.22% Mg as concentrations of these elements are much lower in crustacean flesh, and also exhibiting a wide range of concentrations from 110 to 5640 ppm for Ca, and from 72 to 7670 ppm for Mg (for comparison, see Table 5).

**Table 5:** Comparison of Ca, Mg, Na, K, and Fe concentrations in crabs from different regions

Species name	Habitat	Metals				
		Ca	Mg	Na	K	Fe
		ppm				
*Paratelphusa lamellifrons* ^a^	Padma River, Bangladesh	5385–5640	–	–	1037–1192	423–487
*Uca tangeri* ^b^	The Cross River, Nigeria	7180	7670	6300	6060	1560
*Callinectes amnicola* ^b^	The Cross River, Nigeria	4020–5640	1250–1800	2600–4410	2000–3610	980–1710
*Podopthalmus vigil* ^c^	Parangipettai, India	1567	–	3010	4780	3570
*Callinectes amnicola* ^d^	Ojo River, Nigeria	800–900	–	–	–	690–820
*Cardisoma rotundum* ^e^	Bay of Bengal, India	2168	765	1024	1369	671
*Callinectes sapidus* ^f^	Akyazan Lagoon, Turkey	3982–4554	744–1170	6639–11,330	628–691	–
*Calappa lophus* ^g^	Parangipettai, India	2346–2452	786–1609	1114–1488	1467–1632	64.5–68.6
*Callinectes sapidus* ^h^	The Acquatina Lagoon, Italy	1196	3500	1883	3020	3
*Eriphia verrucosa* ^h^	The Acquatina Lagoon, Italy	4567	6600	3259	2784	4.6
*Cancer pagurus* ^h^	The Acquatina Lagoon, Italy	1286	4200	2120	3881	5.7
*Podopthalmus vigil* ^i^	Parangipettai, India	114–145	9.9–25.0	115–158.9	–	74.5–133
*Charybdis natator* ^j^	Parangipettai, India	115–234	182–688	364–523	248–453	10–25
*Sudananautes africanus* ^k^	River Osun, Nigeria	279–330	222–283	207–260	153–300	71–97
*Eriphia verrucosa* ^l^	Sinop coast, Turkey	8968	1014	23,000	30,350	129
*Cancer pagurus* ^m^	Scottish coast	200–19,260	72–900	1100–18,780	1080–4880	3.2–45

^a^Islam et al. (2016)

^b^Jimmy and Arazu (2012)

^c^Sudhakar et al. (2011)

^d^Moronkola et al. (2011)

^e^Silambarasan et al. (2015)

^f^Küҫükgülmez et al. (2006)

^g^Kathirvel et al. (2014)

^h^Zotti et al. (2016)

^i^Soundarapandian et al. (2014)

^j^Soundarapandian et al. (2013)

^k^Adeyeye et al. (2010)

^l^Erdem et al. (2015)

^m^Barrento et al. (2009)

In our study, high concentrations were also recorded for Na, K, and Fe (8610 ppm, 3780 ppm, and 1830 ppm, respectively). The levels of these elements in crustaceans may occur in broad ranges, as shown in Table 5, ranging from 115 to 23,000 ppm for Na; from 153 to 4880 ppm for K, and from 3 to 1710 ppm for Fe (Table 5). Their concentrations in living organisms are usually high due to their significant role in physiological functions, and their high variability is related to various factors, e.g., sexual dimorphism or the season of catch (Jimmy and Arazu 2012; Islam et al. 2016). The seasonal and sex-specific differences are most probably related to the spawning cycle and differences in metabolism, influencing feeding, reproductive state, and weight (Legras et al. 2000).

The concentration of Fe in the Harris mud crab was about 22% higher than in the bottom sediments, which were also rich in this metal (1430 ppm, Table 3). Despite this significant difference (*p* = 0.02957), it can be concluded (after Rainbow 2007) that the adsorption of Fe from sediments on the surface of the carapace could be a significant factor responsible for its concentration in the crab’s body. The carapace is made of chitin, which has a high sorptive capacity for metals (Rana et al. 2009). This process could also have been behind the relatively high concentrations of Al and Ni in the Harris mud crab (the concentrations in sediments were higher than in the crab, more than 3 times for Al, and more than 2 times for Ni).

The next in terms of concentration were Zn and Cu (62.6 ppm and 25 ppm, respectively) which did not exceed levels recorded in other species of crabs (see Table 6). The concentrations of Zn and Cu in the Harris mud crab were higher than in the bottom sediments (more than 20 times for Zn and about 15 times for Cu). A similar disproportion between Zn and Cu levels in crustaceans and bottom sediments was demonstrated by MacFarlane et al. (2000) in the Semaphore crab (*Heloecius cordiformis*). These metals accumulated in the body of crabs, and especially in the liver. For example, Cu in the liver of *H. cordiformis* ranged from 200 to 600 ppm despite Cu concentrations in sediments ranging from 0.6 to 135 ppm (MacFarlane et al. 2000).

**Table 6:** Comparison of essential and non-essential metals concentrations in crabs from different regions

Species name	Habitat	Cu	Zn	Pb	Cd	Al	Ni
		ppm					
*Xenograpsus testudinatus* ^a^	Kueishan Island, Taiwan	53–290	119–610	1.83–2.64	0.49–1.29	–	0.95–4.76
*Eriocheir sinensis* ^b^	Odra Estuary, Poland	3.17–21.9	1.15–26.6	0.29–0.61	0.016–0.025	2.64–3.49	0.28–2.71
*Rhithropanopeus harrisii* ^c^	Gujarat Purna Estuary, India	6.4–37.1	32–192	–	–	–	16–134
*Charybdis longicollis* ^d^	Iskenderun Bay, Turkey	77.5–935	32.5–805	–	25.4–111	–	–
*Scylla serrata* ^e^	Dapeng Bay, Taiwan	68.98	227	0.06	0.18	–	1.03
*Portunus pelagicus* ^e^		9.9–26.2	39.9-48.7	1.28	0.41	–	0.45–0.78
*Thalamita crenata* ^e^		6.6–25.1	43.6-50.5	0.16–0.83	0.05–0.30	–	0.16–0.39
*Scylla serrata* ^e^		56.8–192	17.5–78.5	1.34–3.70	1.22–2.71	–	1.04–1.70

^a^Peng et al. (2011)

^b^Nędzarek et al. (2017)

^c^Dange and Manoj (2015)

^d^Firat et al. (2008)

^e^Wu (2006)

Zn and Cu are classified as heavy metals essential for animal metabolism in lower concentrations, yet toxic in excess (Rainbow 1985, 2007). Invertebrates use physiological and biochemical detoxification processes that allow them to regulate Zn and Cu concentrations and accumulate them in tissues to survive high environmental concentrations of these metals (Rainbow 1985; MacFarlane et al. 2000; Firat et al. 2008). This ability is non-existent with respect to non-essential metals, such as Pb or Cd, which accumulate permanently in tissues and also then exhibit toxic properties (Rainbow 2007).

Comparing with literature data (see Table 6), the concentrations of Pb (0.14 ppm) and Cd (0.0017 ppm) recorded in our research can be classified as low, considerably lower than the Standard of Maximum Levels of Contaminants in Foods—for both elements maximum 0.50 ppm (FAO 2003, Commission Regulation No. 1881/2006). These metals in the studied crabs could originate from sediments (containing 5 times more Pb and 4 times more Cd than the examined crustaceans). The bioavailability and uptake of non-essential elements by crustaceans depends on different environmental factors such as temperature, salinity, chelating agents, as well as the concentration of these metals in the environment. As benthic organisms have close contact with sediment particles, they accumulate heavy metals via adsorption and food intake (MacFarlane et al. 2000; Firat et al. 2008; Dange and Manoj 2015; Nędzarek et al. 2017; Pires et al. 2017).

**Fig. 2:**
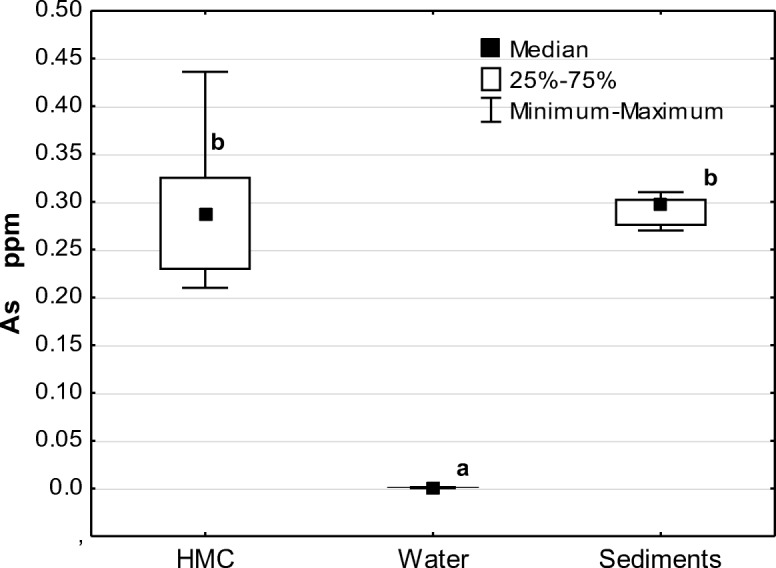
Variance analysis ANOVA (*p* < 0.05) of variation in arsenic distribution in Harris mud crab (HMC), water, and sediments; a, b values marked with the same index do not statistically significantly differ

Bottom sediments could also have been a source of As in the studied crabs, which reached a concentration of 0.288 ppm, not significantly different (*p* = 1.0000) from sediment concentrations (0.291 ppm), but 300 times higher than in the water (Fig. 2). The sediment concentrations of As were lower than those recorded in deep-water zones of the Baltic Sea. For example, in Bornholm Deep (depth 118 m) sediment As concentration was 17 ppm, in Gotland Deep (depth 118 m) 13.3 ppm, in the Gulf of Gdańsk (depth 52 m) 5.1 ppm. A similarly low sediment concentration of As (5.7 ppm) was recorded in Słupsk Farrow (depth of 31 m; distance to the shore, 32 km), the region nearest to our research area (Bełdowski et al. 2016). This difference allows us to conclude that the studied crabs caught in the coastal region were not exposed to elevated concentrations of this potentially toxic element. As a consequence, its accumulation was lower than the recorded concentrations in various crab species by other authors (see Table 7) and did not exceed the International Standards for Maximum Levels of As concentrations in food.

**Table 7:** Comparison of As concentrations in crabs from different regions

Species	Habitat	As
		ppm
*Rhithropanopeus harrisii* ^a^	South Baltic Sea, Poland	0.288 ± 0.063
*Eriocheir sinensis* ^b^	Odra Estuary, Poland	0.035–0.111
*Portunus pelagicus* ^c^	The Persian Gulf, Iran	0.08–0.22
*Portunus pelagicus* ^d^	Gulf of Thailand, Thailand	4.75 ± 2.10
*Uca tangeri* ^e^	Guadalquivir Estuary, Spain	1.76 ± 0.08
King crab^f^	Norwegian surveillance program	26.0 ± 3.0
Crab^g^	Bo Sea, China	0.76 ± 0.02

^a^Own data

^b^Nędzarek et al. (2017)

^c^Khoramnejadian and Fatemi (2015)

^d^Ruangwises and Ruangwises (2011)

^e^Suner et al. (1999)

^f^Sloth et al. (2005)

^g^Li et al. (2003)

The reported concentrations of Cd, Pb, and As in the Harris mud crab were also below the acceptable concentrations of these elements in feed as standardized by Directive 2002/32/EC, according to which feed materials of animal origin may contain a maximum of 2 ppm Cd, 10 ppm Pb, and 25 ppm As.

### Summary

The waters in the survey area and bottom sediments can be considered poorly contaminated with non-essential elements and the bioavailability of Al, Ni, Cd, and Pb for Harris mud crabs is low.

The obtained results show that the Harris mud crab is rich in Ca, Mg, Na, K, Fe, and essentials metals such as Cu and Zn, and can be a valuable source of these elements. The low concentrations of non-essential metal such as Pb, Cd, and As will not pose a threat to consumers.

We can therefore conclude that the invasive Harris mud crab from this Baltic region can be used to prepare well-balanced feed for fish and other animals, and that the introduction of commercial fishing of this crab could help to reduce its population.
